# I Didn’t Know

**Published:** 1913-08

**Authors:** 


### "I Didn't Know."

It was a certain dark December morning in Boston some fifteen years ago. There had been a heavy snowstorm, and the little crooked streets of the North End were choked with grimy snowbanks, that, melting, spread an icy film over the uneven brick pavement. The day was gray and lowering; there was not a gleam of cold sunshine to strike a sparkle from the icicles dripping along the eaves of the high pitched roofs of the old houses—roofs which had once sheltered the dignity and intelligence and integrity of Boston. The houses, with their pillared doorways and curving, wrought-iron handrails, still had dignity, in spite of the squalid uses of adversity; but the human lives, huddled in the stately, dirty old rooms, like rabbits in a warren, had only the dignity of the elemental passions—love and grief and the desire for life. As for intelligence, there was little enough of that in the whole run-down locality. And without intelligence one need hardly look for integrity.

From one of these old houses, swarming with tenants, had come early that morning an appeal; it was written in lead pencil, on a crumpled scrap of paper, and it was very brief:

"Pleas com an help us, for Mamie's sak. She's in troubel."

When I reached No. 42, where "Mamie's" family rented one room which they called "home," I pushed open the battered, unlatched front door under its leaded fanlight, and went up the staircase.

There were four persons in the room; a mother, gaunt and heavy-eyed, sitting with her worn hands for once idle in her lap: she was rocking mechanically back and forth; sometimes she spoke softly to herself; sometimes a tear trickled down her face. Standing opposite her, one foot on a chair, his elbow on his knee, his unshaven chin on his fist, was the father. He spoke only once, and then it was to swear at a boy of nearly 16, who stood in sulky silence between them. This boy was turning his cap around and round his hands; occasionally he kicked steathily at the braided red and black rug in front of the stove; he would not look at either the father or the mother; nor would he look at a little girl who stood beside him. She was just 14; her skirts were still short, her hair in two braids down her back; her thin, childish hands were twisting together; once or twice she glanced at her father and mother, and the lines of bewildered fright in her small sick face sharpened curiously. She shuffled from one foot to the other, and her lips contracted with pain.

Her mother, without looking at her, said dully, and with evident effort, "Better sit down, Mamie." Then turning to me she added drearily, "Her baby'll be born next month. Yes—that boy there, he's the father."

"I'd like to take the hide off him," said the man, under his breath.

"His folks feel 'bout as bad as we do over it," the woman said, as if trying to be just; "they're nice folks, real genteel; they live on the first floor. His father give him a whalin'—but there! What was the use? The trouble's made."

In blank dismay I sat staring at the unhappy group.

Help them! Could any "help bring back to the father and mother their lost opportunity? Could it save a child of 14 from the responsibilities of maternity? How was she to be helped to live, to suffer, to bear the solemn, human burden of giving life? Not by reproaches, certainly; the terrified, sick girl did not know what was happening to her. When the mother suddenly broke out into piercing ejaculations of shame, Mamie only said, in a faint, frightened voice, "I didn't know—" Nor could anything be accomplished by approaching the sulky, ignorant boy, who, with a show of impudence to hide his fear, made the same response: "Well, I wasn't thinking that—that anything would happen; I didn't know—" One could not appeal to shame,—these children did not know the meaning of the word; there can not be shame—cleansing, cauterizing, saving shame—unless there is knowledge of righteousness; no, the shame was not for the children—it was for the parents, for they "knew," and had never shared their knowledge! Nor was it a moment to talk of sin—to the children.

These two poor babies were not sinners; *they were as far from sin as they were from virtue;* they were simply two little pleasant animals, unmoral, as all animals are; and their untrained instinct had led them into a situation in which is rooted the deepest moralities of the race: the relation of father and mother to another soul. It would have been absurd to classify as "wicked" the race-impulse, which becomes moral or immoral only when knowledge is added to it. No; the children were not to blame. As for the father and mother, that is another story.

It was this scene of childish fright and pain, and of helpless adult anger and dismay, that came into my mind when I read Judge Lindsey's article published in the January number of the *Ladies' Home Journal.* And I knew that not one word of its warning was exaggerated. To be sure the reply may be made that in Mamie's walk of life girls are especially defenseless; that children of more fortunate birth would not need the protection of knowledge to keep them from the results of joyous, unmoral animalism. Of course, it is true that Mamie's unguarded poverty did leave her peculiarly undefended. But that was not why this baby of 14 was going to bring another baby into her bitter world of penury and toil. It was because, as she so pitifully reiterated, she "didn't know." When this same statement is made by girls in a better class—girls whose fathers are clerks, business men, professional men—one stands appalled at the amount of avoidable misery which crashes into family life. Ignorance! Ignorance! Not viciousness. Very few children are vicious; they have no more wickedness than puppies; but they have the instinct of puppies—and demi-gods! They have the creative instinct, which, informed, becomes solemn and beautiful and tender, and makes us little lower than the angels; but, uninformed, that same instinct may drag us far below the puppies.

And what of the more guarded children—the children whose fathers and mothers do not belong to the class that finds the subject of life either jocose or shameful? Being guarded, these children may be saved from degradation of the body; but what of the unguarded mind? The school gossip of well-brought-up boys is the answer to that—gossip that spills over into girls' ears so that the soul is left unclean.

One hesitates to generalize on such a subject, but I think I may say that, of more than one hundred girls (most of them mothers before they were twenty, of illegitimate children) who have told me more or less of their wretched story, 90 per cent "didn't know."

What are we to do? Certainly we are not to wait to let the children "know" by the inexorable teaching of experience, as poor Mamie knew. We are to take very solemnly to our consciences this fact: *that fathers and mothers are stewards of the mystery of life.* It is for them to keep the mystery sacred in their children's minds by the defense knowledge; by the honor and dignity and tenderness of truth!—*National Congress of Mother's Magazine.*

